# Expression characteristics of *FHIT*, *p53*, *BRCA2* and *MLH1* in families with a history of oesophageal cancer in a region with a high incidence of oesophageal cancer

**DOI:** 10.3892/ol.2014.2682

**Published:** 2014-11-07

**Authors:** ZHIWEI CHANG, WEIJIE ZHANG, ZHIJUN CHANG, MIN SONG, YANRU QIN, FUBAO CHANG, HAIYUN GUO, QINGLI WEI

**Affiliations:** 1 Department of Oncology, The First Affiliated Hospital of Zhengzhou University, Zhengzhou, Henan 450052, P.R. China; 2 Department of Thoracic Surgery, Linzhou Central Hospital, Linzhou, Henan 456550, P.R. China

**Keywords:** esophagus cancer, positive family history, fragile histidine triad, breast cancer type 2 susceptibility protein, mixed lymphocytic-histiocytic lymphoma, genetic predisposition

## Abstract

The aim of the present study was to determine the changes to the expression levels of fragile histidine triad (*FHIT*), breast cancer type 2 susceptibility protein (*BRCA2*), MutL homolog 1 (*MLH1*) and tumour protein 53 (*p53*) exhibited by families with a history of oesophageal cancer in a region that has a high incidence of oesophageal cancer, and to determine the association of these changes with the cancer history of the families. Immunohistochemistry was used to detect the protein expression of FHIT, p53, BRCA2, and MLH1 in the excised specimens of cancer tissues from 74 oesophageal cancer patients (positive family history of oesophageal cancer [OCFH +], n=33; negative family history of oesophageal cancer [OCFH −], n=41) from a region with a high incidence of oesophageal cancer. The positive expression rates of *FHIT* (61%; 45/74), *BRCA2 (*50%; 37/74) and *MLH1* (27%; 9/33*)* in the oesophageal cancer tissues were significantly lower than those in the healthy tissues adjacent to the cancer (97% [29/30], 87% [26/30] and 73% [25/41], respectively). A significant difference was identified between the positive expression rates (P<0.01). However, *FHIT*, *p53*, *BRCA2* and *MLH1* expression demonstrated no significant affect on clinicopathological changes, such as oesophageal cancerous tissue differentiation, the degree of infiltration and cancer cell metastasis. The *FHIT*, *BRCA2* and *MLH1* expression levels were identified to be significantly lower in the cancer tissues from OCFH + patients. This result indicates that the expression levels of FHIT, BRCA2, and MLH1 are important molecular indices of genetic susceptibility to oesophageal cancer.

## Introduction

Linzhou is a city in the province of Henan in China, that has the highest oesophageal cancer morbidity and mortality rate in the world (1–3). Oesophageal cancer patients in Linzhou have an evident familial aggregation phenomenon. The family members of oesophageal cancer patients are associated with a markedly higher risk of prevalence (4). A descendant of a family with a positive history of oesophageal cancer (OCFH +) tends to have a higher tumour susceptibility when compared with a descendant of a family that has a negative history of oesophageal cancer (OCFH −); the susceptibility increases with the positive family history (5–7). This observation indicates that genetic factors may be significant in the occurrence of oesophageal cancer; however, the molecular basis for susceptibility to this disease remains unclear. A previous study has shown that chromosomal instability and dysfunction in the DNA damage repair mechanism may be involved (8). Fragile histidine triad (*FHIT*) is a recently identified candidate tumour suppressor gene, which is located on the most active site of the human genome (position 3P14.2) and studies demonstrated that the expression of *FHIT* is reduced or absent in various malignant tumours (9,10). MutL homolog 1 (*MLH1*) and breast cancer type 2 susceptibility protein (*BRCA2*) are key genes involved in DNA damage repair, particularly when the damage is induced by foreign carcinogenic factors (11,12). Furthermore, these genes are closely associated with chromosomal stability. Variation in the tumour protein 53 (*p53*)-retinoblastoma protein system is a common molecular event (13) that is observed in oesophageal cancer patients from Henan, where there is a high incidence of oesophageal cancer. In the present study, the expression of the fragile site genes, *FHIT* and *p53*, which are associated with chromosomal stability, were analysed in excised oesophageal cancer tissue specimens, as well as the DNA damage repair genes, *BRCA2* and *MLH1*. The association between these genes and the family history of cancer was also determined. The results of the present study may contribute to understanding the molecular mechanism of, and genetic susceptibility of humans to, oesophageal cancer in areas with a high incidence of this type of cancer.

## Patients and methods

### Patients and samples

Of the subjects enrolled in the present study, 33 were OCFH + and 41 were OCFH −; all of the subjects were residents of Linzhou (Henan, China). OCFH + patients had a minimum of two family members who suffered from the disease and were descendants of families who lived in a region with a high incidence of oesophageal cancer, in which two generations had suffered from the disease (8). OCFH − patients were those who had no family members who had suffered from the disease or from other types of cancer. Of the OCFH + patients, 24 (73%) were male and nine (27%) were female. The patients were aged between 46 and 72 years, with an mean age of 56±9 years. Of the OCFH − patients, 26 (63%) were male and 15 (37%) were female. The patients were aged between 35 and 71 years, with an average age of 56±9 years. None of the patients had received chemotherapy or radiotherapy prior to radical resection. Following surgery, the cancer specimen was immediately fixed with 85% alcohol, dehydrated via routine histology, embedded in paraffin and sliced into 5-μm serial sections. Subsequently, the specimens were classified as well-, moderately or poorly differentiated squamous cell carcinomas, according to the cellular morphology, tissue structure and differentiation. The diagnostic criteria were based on a previous study (13,14) and the clinical pathological conditions of the patients are presented in Table I. Normal adjacent tissue was also obtained, which was ≥5 cm from the carcinoma tissue, as a control. These healthy specimens were confirmed to have no precancerous lesions by pathological examination. Written informed consent was obtained from all patients and the study was approved by the ethics committee of the First Affiliated Hospital of Zhengzhou University (Zhengzhou, China).

### Immunohistochemical detection

The specimen was analysed using the avidin-biotin horseradish peroxidase complex (ABC) method as follows: The sample was embedded in paraffin, sliced, dehydrated in graded alcohol and washed three times with phosphate-buffered saline (PBS) for 5 min. BRCA2 and FHIT antigens were retrieved via microwave irradiation for 10 min. MLH1 was placed in boiling water for 30 min and then cooled to room temperature. Subsequently, 0.5% H_2_O_2_ was added to MLH1 at room temperature for 20 min and the resulting mixture was washed three times with PBS for 5 min. BRCA2 rabbit anti-human polyclonal antibody was purchased from Boster Biological Engineering Co., Ltd., (Wuhan, China; dilution 1:100), MLH1 rat anti-horse monoclonal antibody was purchased from BD Pharmingen (San Diego, CA, USA; dilution, 1:50), the FHIT polyclonal rabbit anti-goat antibody was purchased from Beijing Zhongshan Biotechnology Co., Ltd. (Beijing, China) (ZA-0410; 1:100 dilution) and p53 rat anti-horse monoclonal antibody (1:1,000) was purchased from Nuclea Biotechnologies, Inc., (Pittsfield, MA, USA). The mixture was then incubated with normal horse or sheep serum at room temperature for 20 min and the primary antibody was added. FHIT, BRCA2 and MLH1 were diluted with 2% bovine serum albumin (BSA) at ratios of 1:100, 1:100 and 1:50, respectively. The mixtures were placed in a humidified chamber, maintained for 12 h in a freezer at 4°C and washed three times with PBS for 5 min. A secondary antibody [horse anti-rat, binding to the rat antibodies of MLH1 and p53, or goat anti-rabbit, binding to the rabbit antibody of BRCA2 and FHIT; dilution 1:200; Thermo Fisher Scientific, Waltham, MA, USA) was added to the samples and the mixtures were then incubated for 45 min (2% BSA dilution; 1:200) and washed three times with PBS for 5 min. Subsequently, the samples were incubated in ABC (that had been prepared 30 min prior to use) for 60 min and washed three times with PBS for 5 min. The samples were then incubated in 3,3′-diaminobenzidine (Vector Laboratories Inc., Burlingame, CA, USA) and H_2_O_2_, and observed under a microscope (BX53T-32P01, Olympus Corporation, Tokyo, Japan). Following this, the reactions were terminated in a timely manner. The samples were stained with haematoxylin for 15–30 sec, observed under a microscope (BX53T-32P01, Olympus Corporation), dehydrated in graded alcohol and processed with xylene to produce transparent slices, which were mounted with neutral gum (Nanjing Shenglide Biological Technology Co., Ltd., Nanjing, China).

The negative control consisted of the sera of normal animals that produce the secondary antibody that blocks non-specific immunoglobulin responses; this sample did not contain the primary antibody. A biopsy sample that was known to be positive for MLH1, FHIT, p53 and BRCA2 served as the positive control.

### Result determination

Each slice was observed under a high-power lens (BX53T-32P01; magnification, ×40) and a minimum of five random fields were counted to obtain the mean number of positive cells. Immunohistochemical staining results for FHIT and BRCA2 were determined based on the semiquantitative method described by Greenspan *et al* (10). The staining intensity scoring was conducted as follows: 1, Lack of expression or weak expression; 2, moderate expression; and 3, strong expression. The positive cell classification was determined as follows: 1, <10% of cells were positive; 2, 10–50% of cells were positive; and 3, >50% of cells were positive. The final score was obtained by multiplying the two scores. An FHIT score of ≤3 points indicated decreased or absent expression and was considered to be negative immune expression, whereas a score of ≥3 indicated a positive immune expression. A BRCA2 score of <3 points indicated reduced expression and was considered to be negative immune expression, whereas a score of >3 indicated a positive immune expression. For MLH1, a percentage of <10% positive cells was considered to be negative immune expression, whereas a value of ≥10% positive cells was considered to indicate positive immune expression (14). With regard to p53, the appearance of three or more brown-yellow or brown cell nuclei under high-power (magnification, ×400) was considered to be positive immune and positive protein expression (13).

### Statistical analysis

The results were processed using the SPSS 13.0 statistical software. The *χ*^2^ and Spearman’s correlation tests were used to determine the correlation between clinical pathology and protein expression data. P<0.05 was considered to indicate a statistically significant difference.

## Results

### Immunohistochemical detection of FHIT, p53, BRCA2 and MLH1

**Figure 1:**
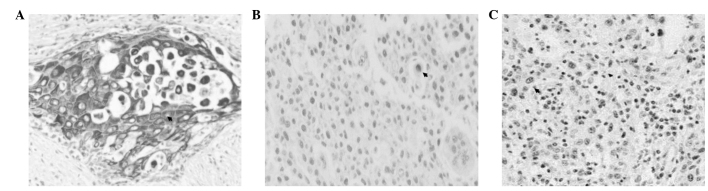
Fragile histidine triad (FHIT) immune response in oesophageal carcinoma tissues. (A) Strong and positive reaction for FHIT expression. Immunoreactivity was predominantly located in the cytoplasm and nucleus; a positive cancer cell is shown by the arrow. (B) Weak and positive reaction for FHIT expression. Immunoreactivity was predominantly located in the cytoplasm and nucleus; a positive cancer cell is demonstrated by the arrow. (C) Negative reaction for FHIT expression; a negatively expressed cell is shown by the arrow. Magnification, ×400; haematoxylin and eosin restained.

**Figure 2:**
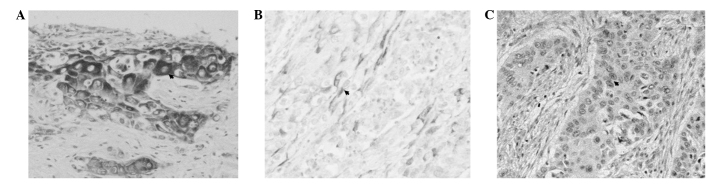
Breast cancer type 2 susceptibility protein (BRCA2) immune response in oesophageal carcinoma tissues. (A) Strong and positive reaction for BRCA2 expression. Immunoreactivity was predominantly located in the cytoplasm and cell membrane; a positive cancer cell is shown by the arrow. (B) Weak and positive reaction for BRCA2 expression. Immunoreactivity was predominantly located in the cytoplasm and membrane; a positive cancer cell is shown by the arrow. (C) Negative expression for BRCA2; a negatively expressed cell is shown by the arrow. Magnification, ×400; haematoxylin and eosin restained.

**Figure 3:**
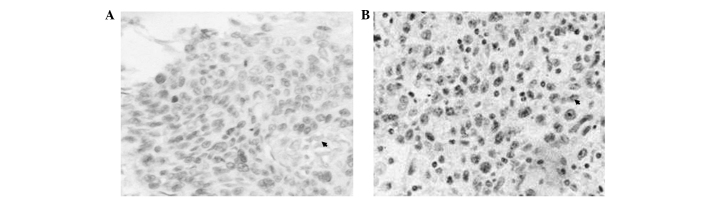
Mixed lymphocytic histiocytic lymphoma (MLH1) immune response in oesophageal carcinoma tissues. (A) Positive reaction for MLH1 expression. Immunoreactivity was predominantly located in the nucleus; a positive cancer cell is shown by the arrow. (B) Negative reaction for MLH1 expression; a negatively expressed cell is shown by the arrow. Magnification, ×400; haematoxylin and eosin restained.

FHIT immunoreactivity was predominantly detected in the cytoplasm and nucleus and visualised as black coloration (Fig. 1). BRCA2 immunoreactivity was predominantly observed in the cytoplasm and membrane as black coloration (Fig. 2), and MLH1 and p53 immunoreactivities were primarily detected in the nucleus as black coloration (Fig. 3).

### Analysis of FHIT, MLH1, BRCA2 and p53 expression levels

The positive expression rate of *FHIT* (61%; 45/74) in the oesophageal cancer tissues was identified to be lower than that of the adjacent healthy tissues (97%; 29/30) and the difference was statistically significant (P<0.01). The positive expression rate of *BRCA2* (50%; 37/74) in the oesophageal cancer tissues was markedly lower than that in the adjacent healthy tissues (87%; 26/30), and the difference was identified to be statistically significant (P<0.01). Furthermore, the positive expression rate of *p53* in the oesophageal carcinoma tissues of the OCFH + patients (52%; 17/33) was greater than that of the OCFH − patients (46%; 19/41), although the difference was not statistically significant (P>0.05). The positive expression rate of *FHIT* in the cancer tissues of the OCFH + patients (46%; 15/33) was significantly lower than that of the OCFH − patients (73%; 30/41), and the difference was statistically significant (P<0.05; Table IV). The positive expression rate of *BRCA2* in the oesophageal carcinoma tissues of the OCFH + patients (33%; 11/33) was lower than that of the OCFH − patients (63%; 26/41) and the difference was statistically significant (P<0.05). The *MLH1* positive expression rate in the cancer tissues of the OCFH + patients (27%; 9/33) was lower than that of the OCFH − patients (61%, 25/41) and the difference was statistically significant (P<0.01; Table V).

### Correlation analysis of FHIT, BRCA2 and MLH1 expression levels

No significant correlation was observed between the positive expression rates of *FHIT* (46 and 73%) and *BRCA2* (33 and 63%), between the positive expression rates of *FHIT* (46 and 73%) and *MLH1* (27 and 61%), and between the positive expression rates of *FHIT* (46 and 73%) and *p53* (52 and 46%) in the oesophageal cancer tissues of all patients. Furthermore, no significant correlation was identified between the positive expression rates of *BRCA2* (33 and 63%) and *MLH1* (27 and 61%), between the positive expression rates of *BRCA2* (33 and 63%) and *p53* (52 and 46%), and between the positive expression rates of *MLH1* (27 and 61%) and *p53* (52 and 46%) (P>0.05; Tables IV–VII).

### Associations between MLH1, BRCA2, and FHIT expression levels and the clinicopathology of oesophageal carcinoma

The *BRCA2* negative expression rate gradually decreased in the well− (60%; 6/10), moderately (46%; 22/48) and poorly (56%; 9/16) differentiated cancer tissues, however, the differences between results were not significant (P>0.05). The negative expression rates of *MLH1* in the well−, moderately, and poorly differentiated oesophageal cancer tissues were 80% (8/10), 48% (23/48) and 56% (9/16), respectively. These values initially decreased and subsequently increased, although the changes were not statistically significant (P>0.05). The negative expression rates of *FHIT* in the well−, moderately, and poorly differentiated carcinoma tissues were 40% (4/10), 38% (18/48) and 44% (7/16), respectively. The negative expression gradually decreased (P>0.05). The negative expression rates of *p53* in the well−, moderately, and poorly differentiated carcinoma tissues were 50% (5/10), 52% (25/48) and 56% (9/16), respectively. However, the difference was not statistically significant (P>0.05).

The expression levels of *BRCA2*, *FHIT*, *MLH1* and *p53* in the lymph node metastasis group showed no statistically significant difference with those of the lymph node non-metastasis group. As the extent of tumour invasion increased (mucosa and submucosa to muscularis to adventitia), the negative expression rates of *BRCA2* and *FHIT* gradually decreased (67 to 45 to 44% vs. 56 to 35 to 33%), the *p53* negative expression rates gradually decreased (56 to 50 to 50%), and the *MLH1* negative expression rates initially decreased and then increased (78 to 45 to 47%). However, these changes were not statistically significant (P>0.05). In male patients, the negative expression rate of *BRCA2* and the negative expression rate of *p53* were marginally higher when compared with female patients (52 and 46 vs. 54 and 46%), whereas the *FHIT* negative expression rate was marginally lower than that of the females (36 vs. 46%). However, the differences were not statistically significant (P>0.05). The negative expression rates of *MLH1* in males and females showed no statistically significant difference (Tables VII).

## Discussion

The high incidence of oesophageal cancer within families is a common phenomenon in geographical regions with a high incidence of oesophageal cancer, with members of families with a history or oesophageal cancer being associated with a higher risk of prevalence. A previous study demonstrated that various fragile sites are present in the members of families with a high incidence of oesophageal cancer (8). Approximately 89% of subjects of the high incidence families were carriers of fragile sites (primarily the common fragile sites), which were vertically inherited according to the Mendelian monogenic autosomal recessive model of blood relatives (15). Therefore, oesophageal cancer may be associated with a familial predisposition to fragile sites, as well as chromosomal instability (8). In oesophageal cancer patients and their children, the rates of chromosomal aberrations and appearance of fragile sites significantly increased, when compared with healthy individuals (16). Furthermore, the consistent compliance rates of fragile sites, oncogenes and cancer breakpoints in oesophageal cancer patients were also significantly higher than those in the control group. This result indicates that chromosomal instability increases the susceptibility of an individual to cancer and may be the genetic basis of oesophageal cancer (16).

In the present study, OCFH + patients showed significantly higher negative expression rates of *FHIT*, *BRCA2* and *MLH1* when compared with the OCFH − patients. These results indicate that *FHIT* may be involved in the genesis of oesophageal cancer and is possibly closely associated with the high susceptibility of family members in the region of Linzhou, of high oesophageal cancer incidence. In addition, the *BRCA2*, *MLH1* and *FHIT* genes may be involved in the genesis of oesophageal cancer in susceptible populations, and the abnormal changes in *BRCA2* and *MLH1* expression are important molecular events in the occurrence of oesophageal cancer in OCFH + patients. These proteins may be significant molecular bases for the genesis of a high susceptibility of individuals to oesophageal carcinoma. In the present study, the positive expression rates of *p53* were markedly high in all of the oesophageal cancer patients, regardless of the family medical history. Although the rates are higher in the OCFH + group, the difference was not significant, which indicates that *p53* is associated with additional predisposing factors that exert synergistic effects on oesophageal cancer susceptibility. Thus, the association between *p53* and a high susceptibility to oesophageal cancer requires further investigation. As an identified tumour suppressor gene, *FHIT* is positioned on the third chromosome (3p14.2), which contains the majority of the active fragile sites of the human genome, as well as numerous chromosomal abnormalities. A chromosomal exception that occurs in this site commonly leads to *FHIT* inactivation and abnormal protein expression (9). Furthermore, the *BRCA2* gene is located at chromosome 13q12, where a high frequency of allelic loss in tumours occurs (17). Therefore, the BRCA2 protein is essential in maintaining the stability of chromosomes and is involved in the DNA repair process. BRCA2 is activated by *RAD51* and is involved in cell cycle regulation (18). In addition, the *BRCA2* gene 203G>A polymorphism may be associated with susceptibility to oesophageal cancer (19). *BRCA2* mutation causes single- or double-strand break repair defects that lead to chromosomal instability; the incidence of *BRCA2* gene mutations in OCFH + patients was found to be significantly higher than that in OCFH − patients (20). This result demonstrates that *BRCA2* is closely associated with a genetic susceptibility to familial oesophageal cancer. Mismatch repairs primarily indicate an excision and repair process that is directed towards the nucleotide of the contralateral DNA chain. In addition to DNA repair, mismatch repair also transfers DNA damage signals to the apoptosis initiation system; DNA damages that cannot be repaired induce apoptosis (11,12). Additionally, the carcinogenic nitrosamine, methylbenzylnitrosamine inactivates or reduces the expression of mismatch repair genes in oesophageal cancer and inhibits the mismatch repair function of cells, thus increasing cancer risk (21).

Damage to the *FHIT* gene, which is closely associated with chromosomal abnormalities, may result from DNA repair deficiencies (22). Previous studies have shown that *FHIT* expression levels are significantly increased in a number of DNA repair-deficiency tumours, such as breast cancer and colorectal cancer (10,14,21,22). Abnormal conditions, such as chromosome breakage are observed in cell lines with mismatch repair and double-strand break repair gene defects (23). This phenomenon indicates that DNA repair deficiencies significantly affect chromosomal stability and increase the susceptibility of a number of fragile gene sites, such as *FHIT* to abnormalities.

In conclusion, it is hypothesised that individuals who are susceptible to oesophageal cancer may exhibit a high incidence of chromosomal instability. Therefore, the risk of chromosomal abnormality is higher when individuals are affected by the same carcinogenic factors in familiar environments. This condition causes the abnormal expression of a number of key genes (such as oncogenes or cancer suppressor genes) located in certain unstable areas (such as fragile sites), which subsequently leads to earlier carcinoma genesis. In the present study, however, *FHIT* expression in oesophageal cancer patients showed no significant association with *BRCA2* and *MLH1* negative expression regardless of the family medical history. No significant correlation was identified between the negative expression of *BRCA2* and *MLH1*, which indicates that the high susceptibility to oesophageal cancer is a complicated synergy that involves multiple genes. By contrast, the correlation among *FHIT*, *BRCA2* and *MLH1* expression involves simple, causal connections rather than multiple factors. In addition to *BRCA2* and *MLH1*, other important factors also affect *FHIT* expression levels. The synergistic effect of *FHIT*, *BRCA2*, *MLH1* and other relevant factors may be the molecular bases for the genesis of oesophageal cancer. Further discussion of the association between DNA repair, chromosomal stability, and *FHIT*, *BRCA2* and *MLH* expression in susceptible populations may contribute to elucidating the molecular basis for the high susceptibility of oesophageal-cancer families in regions with a high incidence of oesophageal cancer.

## Figures and Tables

**Table I:** Clinical pathology of oesophageal cancer patients with a positive or negative family history of oesophageal carcinoma in Linzhou, China.

		OCFH +	OCFH −
		
Clinical pathology	Cases, n	n (%)	n (%)
Age, years			
≤40	2	2 (6)	0 (0)
41–50	20	10 (30)	10 (24)
51–60	32	15 (46)	17 (42)
>60	20	6 (18)	14 (34)
Gender			
Male	50	24 (73)	26 (63)
Female	24	9 (27)	15 (37)
Infiltration			
Mucosa	7	4 (12)	3 (7)
Submucosa	11	6 (18)	5 (12)
Muscularis	20	10 (30)	10 (25)
Fibrous membrane	36	13 (40)	23 (56)
Differentiation			
Well−	10	7 (21)	3 (7)
Moderately	48	19 (58)	29 (71)
Poorly	16	7 (21)	9 (22)
Metastasis			
Yes	22	6 (18)	16 (39)
No	52	27 (82)	25 (61)
Total	74	33	41

OCFH, oesophageal cancer family history; +, positive; −, negative.

**Table II:** *p53* and *FHIT* expression analysis in patients with a positive or negative family history of oesophageal cancer.

		*p53* + expression	*FHIT* + expression
		
Group	Cases, n	n (%)	n (%)
OCFH +	33	17 (52)^a^	15 (46)^b^
OCFH −	41	19 (46)^a^	30 (73)^b^
Total	74	36	45

*FHIT*, fragile histidine triad; *p53*, tumour protein 53; OCFH, oesophageal cancer family history; +, positive; −, negative.

a P>0.05 vs. OCFH − and

b P<0.05 vs. OCFH −.

**Table III:** *BRCA2* and *MLH1* expression analysis in patients with a positive or negative family history of oesophageal carcinoma.

		*BRCA2* + expression	*MLH1* + expression
		
Group	Cases, n	n (%)	n (%)
OCFH +	33	11 (33)^a^	9 (27)^b^
OCFH −	41	26 (63)^a^	25 (61)^b^
Total	74	37	34

*BRCA2*, breast cancer type 2 susceptibility protein; *MLH1*, mixed lymphocytic histiocytic lymphoma; OCFH, oesophageal cancer family history; +, positive; −, negative.

a P<0.05 vs. OCFH − and

b P<0.01 vs. OCFH −.

**Table IV:** Correlation analysis of *BRCA2*, *p53*, *MLH1* and *FHIT* expression in the cancer tissues of oesophageal carcinoma patients with or without a family history of oesophageal cancer.

		*BRCA2* negative expression	*p53* positive expression	*MLH1* negative expression
		
*FHIT* expression	Cases, n	n (%)	n (%)	n (%)
OCFH +				
Negative expression	18	13 (72)	8 (44)	12 (67)
Positive expression	15	9 (60)	9 (60)	12 (67)
OCFH −				
Negative expression	11	1 (9)	6 (55)	5 (46)
Positive expression	30	14 (47)	13 (43)	11 (37)

*BRCA2*, breast cancer type 2 susceptibility protein; *p53*, tumour protein 53; *MLH1*, mixed lymphocytic histiocytic lymphoma; *FHIT*, fragile histidine triad; OCFH, oesophageal cancer family history; +, positive; −, negative. No significant correlation was identified between the two groups. P>0.05.

**Table V:** Correlation analysis of *BRCA2*, *p53* and *MLH1* expression in the cancer tissues of oesophageal carcinoma patients with or without a family history of oesophageal cancer.

		*p53* + expression	*MLH1* − expression
		
*BRCA2* expression	Cases, n	n (%)	n (%)
OCFH +			
Negative expression	22	11 (50)	15 (68)
Positive expression	11	6 (55)	9 (82)
OCFH −			
Negative expression	15	8 (53)	5 (33)
Positive expression	26	11 (42)	11 (42)

*BRCA2*, breast cancer type 2 susceptibility protein; p53, tumour protein 53; MLH1, mixed lymphocytic histiocytic lymphoma; OCFH, oesophageal cancer family history; +, positive; −, negative. No significant correlation was identified between the two groups. P>0.05.

**Table VI:** Correlation analysis of *p53* and *MLH1* expression in the cancer tissues of oesophageal carcinoma patients with or without a family history of oesophageal cancer.

		*MLH1* − expression	*MLH1* + expression
		
*p53* expression	Cases, n	n (%)	n (%)
OCFH +			
Negative expression	16	12 (75)	4 (25)
Positive expression	17	12 (71)	5 (29)
OCFH −			
Negative expression	22	6 (27)	16 (73)
Positive expression	19	10 (53)	9 (47)

MLH1, mixed lymphocytic histiocytic lymphoma; p53, tumour protein 53; OCFH, oesophageal cancer family history; +, positive; −, negative. No significant correlation was identified between the two groups. P>0.05.

**Table VII:** Association between *BRCA2, MLH1*, *FHIT* and *p53* positive expression and clinicopathological characteristics of oesophageal carcinoma.

		Positive expression, n (%)			
		
Clinical pathology	Cases, n	*BRCA2*	*MLH1*	*FHIT*	*p53*
Gender					
Male	50	26 (62)	27 (54)	18 (36)	27 (54)
Female	24	11 (46)	13 (54)	11 (46)	11 (46)
Differentiation					
Well−	10	6 (60)	8 (80)	4 (40)	5 (50)
Moderately	48	22 (46)	23 (48)	18 (38)	25 (52)
Poorly	16	9 (56)	9 (56)	7 (44)	9 (56)
Metastasis					
Yes	22	11 (50)	9 (41)	5 (23)	13 (59)
No	52	26 (50)	43 (60)	24 (46)	25 (48)
Infiltration					
Mucosa/submucosa	18	12 (67)	14 (78)	7 (35)	10 (56)
Muscularis	20	9 (45)	9 (45)	7 (35)	10 (50)
Adventitia	36	22 (44)	17 (47)	12 (33)	18 (50)

*BRCA2*, breast cancer type 2 susceptibility protein; *MLH1*, mixed lymphocytic histiocytic lymphoma; *FHIT*, fragile histidine triad; *p53*, tumour protein 53.
